# Intracystic papillary breast carcinoma with areas of infiltration: Report on two cases

**Published:** 2012-06-30

**Authors:** María del Mar Muñoz Díaz, Silvia Martín Gutiérrez, María Antonia Nieto Gallo, Rosario Noguero Meseguera, Ignacio Rodríguez Prieto

**Affiliations:** a Infanta Cristina University Hospital, Parla, Madrid, España; b Department of Obstetrics and Gynecology; c Department of Pathology; d General and Digestive Surgery Services

**Keywords:** Ductal carcinoma *in situ*, breast cancer, papillary carcinoma, papilloma

## Abstract

Intracystic papillary carcinoma of the breast associated with areas of infiltration is rare in that it constitutes less than 1% of breast cancers. After initial radiological study, these tumors show lesions with little likelihood of malignancy in a high proportion of cases. Two cases of intracystic papillary carcinoma associated with infiltration were diagnosed at the Breast Unit of Hospital Infanta Cristina. In both cases, the reason for consultation arose after palpation of a nodule and the initial radiographic analyses showed lesions with little likelihood of malignancy.

## Abstract (es)

El carcinoma papilar intraquístico de mama asociado a áreas de infiltración, es una entidad infrecuente, que constituye menos de un 1% de los cánceres de mama. Se trata un tumor cuyo estudio radiológico inicial muestra lesiones con baja sospecha de malignidad en una proporción elevada de casos. Presentamos dos casos de carcinoma papilar intraquístico, asociados a infiltración diagnosticados en la Unidad de Mama del Hospital Universitario Infanta Cristina. En ambos casos el motivo de consulta fue la palpación de un nódulo y la aproximación radiológica inicial mostró lesiones con baja sospecha de malignidad.

## Introduction

Papillary lesions of the breast include a variety of tumors ranging from benign papillomas, with or without atypia, to papillary carcinomas. Papillary carcinomas can be divided into invasive and non-invasive forms; the latter forms are in turn subdivided into localized (intracystic papillary carcinoma) and diffuse forms considered to be a variety of ductile carcinoma *in situ* ^1^. Intracystic papillary carcinoma is characterized by papillary growth within a cyst and may be associated with ductile carcinoma *in situ* (46%) or with an infiltrating carcinoma (36%)^2^. The absence of myoepithelial cells in the periphery of the areas of the papillary tumor has been histologically used to define the invasive nature of the injury^3^.

The presence of a palpable nodule of recent onset in an older woman together with a bloody nipple discharge and the ecographic finding of a cystic lesion with ill-defined margins should help guide us to its proper diagnosis^4^. The ecographic diagnosis is not simple unless there is clear invasion, the most frequent finding is that of a cystic lesion with areas of thickened wall or, occasionally, intracystic projections (complicated cyst).

Fine needle aspiration (FNA) should be part of the initial study, as it is a technique with a high rate of false negatives of about 22.0 - 37.8%^5^; therefore, when faced with a suspected diagnosis, the diagnostic study must be completed by means of a core needle biopsy (CNB), and possible excisional biopsy, in order to specify the nature of the lesion.

Two cases of intracystic papillary carcinoma are presented associated with infiltration that was diagnosed in the Breast Unit of the Infanta Cristina University Hospital.

### Presentation of cases

### Case #1

A fifty-year-old patient without prior personal or family history of interest was seen in follow-up at the Breast Unit for a simple cyst in the left subareolar region since 2008. On physical examination, the patient had a nodule of 1.5 cm with elastic consistency, not adhered, at the juncture of the lower quadrants of the left breast.

**Figure 1:**
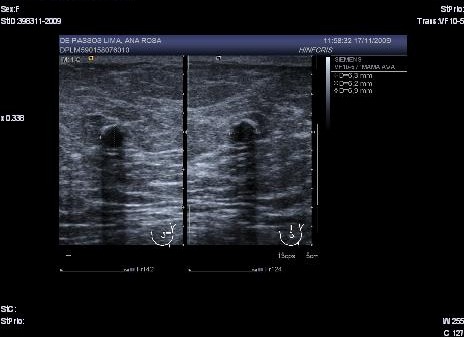
Well-defined cystic lesion with heterogeneous content

In a mammography of April 2009, a subareolar, oval-shaped, well-defined nodule of 16 mm with mammographic characteristics was observed: possibly benign (Birds 0). Extended studies were indicated through ecography of the left breast. The left breast ecography showed a well-defined cystic lesion in the left subareolar region with heterogeneous content, 19.1 x 12 x 17.8 mm that when compared with previous studies since 2006 showed an increase in size (Birads 4). The FNA evidenced a negative extension for malignant cells with findings consistent with the cystic content. With the above results ultrasound monitoring of the lesion after 3 months was decided upon. In the ultrasound of the left breast (July 2009) a cystic lesion was found with heterogeneous subareolar content of 12 x 8.4 x 12 mm (Fig. 1).

**Figure 2:**
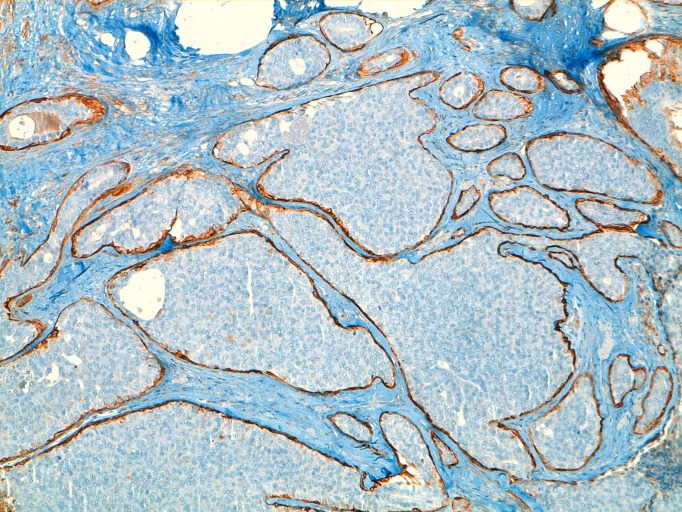
Characteristic labeling of cells with actin HHF35 periphery of nests in areas of in situ component.

Given the findings, a deferred biopsy was decided upon. Pathology reported the existence of a cystic lesion of 13 mm in the lumpectomy sample. Histological sections showed a cystically dilated duct, neoplastic proliferation within an interior of solid and papillary growth with a delicate fibro vascular stroma and small cells with mild atypia and occasional mitotic figures. In the periphery of the cyst tumorous nests were observed consistent with micro-infiltration areas, and confirmation of the absence of myoepithelial cells with techniques of immunohistochemistry (actin and collagen IV). The immunohistochemistry was positive for hormone receptors (estrogen and progesterone), absence of expression of HER-2 and pof 53 negative. The pathological diagnosis was intracystic papillary carcinoma with solid areas of micro-infiltration but without being able to evaluate the margins of the resection (Fig. 2).

The biopsy margins were broadened along with the selective sentinel node, the latter being negative for tumor infiltration. The patient received adjuvant radiotherapy after surgery and hormone therapy with tamoxifen.

### Case # 2

**Figure 3:**
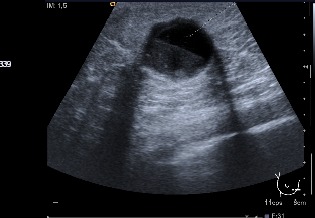
Cyst with content with clearly defined borders

An eighty-one year old patient consulted for an emerging nodule in the right breast without any family or personal history of interest. On physical examination, the patient had a nodule of 20 mm in the lower inner quadrant of the right breast. In the mammogram a nodular image was observed that with ultrasonography was identified as a cyst containing clear borders and good sound transmission (Fig. 3): the FNA evidenced papillary epithelial proliferation.

Given these findings the decision to perform a deferred biopsy was reached. An accidental opening of the cyst occurred intra-operatively along with drainage of a bloody fluid. Pathology reported a well-circumscribed, nodular formation in which a solid proliferation of large nests composed of cells with uniform nuclei was observed with low mitotic activity. This formation had a pattern of expansive growth within a partially collapsed cystic cavity and infiltrating area of ​​10 mm in the wall of the cystic structure. Immunohistochemistry was positive for hormone receptors (estrogen and progesterone), and showed an absence in the expression of HER-2 and p53 negative.

**Figure 4:**
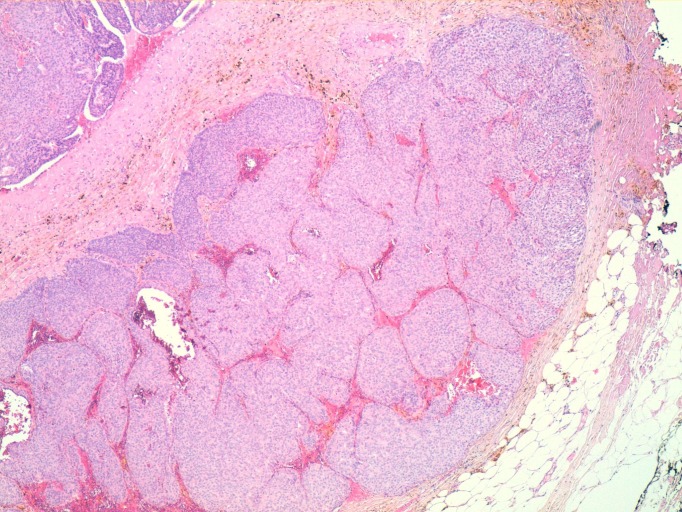
Nests with solid papillary growth pattern. HEx40 technique

The pathological diagnosis was of a solid papillary intracystic carcinoma with a point of invasion - infiltrating papillary carcinoma and intracystic papillary carcinoma (Fig. 4).

The biopsy margins were broadened along with the selective sentinel node, the latter being negative for tumor infiltration. The patient received adjuvant radiotherapy after surgery and hormonal treatment with tamoxifen.

## Discussion

The intracystic papillary carcinoma of the breast is a variety of intraductile papillary carcinoma which may be associated with a ductile carcinoma *in situ* (46%) or an infiltrating carcinoma (36%). It predominantly affects older women, but can also occur in younger women. The average diagnostic age is older than it is for other histological types of breast cancer^6^.

Its clinical expression varies with the presence of a palpable mass, bloody nipple discharge or radiological findings. Age, hemorrhagic content of the cyst (>50%) and the presence of a recurrent cyst or residual mass after FNA require a differential diagnosis with intracystic papillary carcinoma.

As for imaging diagnoses, 98% of mammograms meet the criteria for benignity^7^ showing a well-defined nodule. The most frequent ecographic finding is: a cystic nodule with thickened areas in the wall or, occasionally, intracystic projections (complicated cyst). Magnetic resonance imaging (MRI) may be useful to rule out infiltration.

The FNA should be part of the initial study and with it one can arrive at a diagnosis in 40-80% of cases. If the FNA is insufficient, a core needle biopsy (CNB) will be carried out. In some cases it is necessary to perform an excisional biopsy to arrive at a diagnosis.

Histopathology showed a papillary lesion, sometimes large in size (average 20 mm), located in a large cystic duct, and characterized by thin fibrovascular cores and a population of neoplastic epithelial cells with features of low-grade intraductal carcinoma. It can show a solid growth pattern, cribriform, micropapillary or stratified spindle cells. The absence of a myoepithelial cell layer in the periphery of the papillary tumor reveals the infiltrating nature of the lesion.

The variant of solid papillary carcinoma is characterized by a very dense cellular proliferation that obscures the papillary pattern and the cystic nature of the lesion. They are usually low-grade histological lesions without necrosis and with positive estrogen receptors and absence of HER-2 expression^8^.

Treatment is the same as that for ductile carcinoma *in situ*. Surgery is the first treatment option. If the lesion is associated with infiltration, it should be treated according to the stage of the invasive lesion.

Adjuvant radiotherapy in intracystic papillary carcinomas is not indicated for the forms that are not associated with other types of tumors (extensive ductile carcinoma, invasive carcinoma). Hormone therapy is not justified in the non-invasive forms since it does not modify the prognosis of the disease and yet may increase morbidity by its secondary effects^9^.

Intracystic papillary carcinomas not associated with other types of tumors have a comparatively better prognosis. When not associated with an invasive component, nodal involvement is very low, similar to when it is associated with intraductal carcinoma, but in the latter case it describes an increase in local recurrence. When an infiltrating component is associated, the frequency of axillary metastases is similar to that of infiltrating ductile carcinoma. Despite the good prognosis of this tumor, several authors have described forms of invasive intracystic papillary carcinoma with liver metastases^10^. 

## Conclusions

Intracystic papillary carcinoma is a rare tumor with a very good prognosis which mainly affects older women. Radiological studies usually show lesions with little suspicion of malignancy. The first line of treatment is surgery, and if the lesion is associated with points of infiltration it should be treated according to the stage of the invasive lesion. Adjuvant treatment in the absence of invasion is not justified.
